# Like mother, like daughter, like granddaughter… Transgenerational ignorance engendered by a defective reproductive health technology

**DOI:** 10.1016/j.rbms.2021.10.001

**Published:** 2021-10-30

**Authors:** Emmanuelle Fillion, Didier Torny

**Affiliations:** a Ecole des Hautes Etudes en Santé Publique, ARENES (UMR 5160), Rennes, France; b CNRS, CSI, Mines ParisTech (I3, UMR 9217), PSL University, Paris, France

**Keywords:** Social production of ignorance, Agnotology, Transgenerational effects, Diethylstilbestrol, Reproductive technologies, Drug side effects

## Abstract

From 1941, the synthetic oestrogen diethylstilbestrol (DES) was administered to millions of women around the world to prevent miscarriages. In 1971, a clear and direct link was shown between taking DES during pregnancy and its subsequent long-term morbid effects on offspring. In the last 50 years, the list of side effects of in-utero exposure to DES has grown to include cancer, infertility, significant prematurity and urogenital malformation, amongst others. Based on qualitative sociological research conducted between 2010 and 2013, compiling archives, judicial documents and 108 interviews, this article illustrates a continuous production of ignorance in France. By focusing on DES as a reproductive health technology, three aspects are stressed. First, in terms of recognition of adverse effects, despite DES being identified as a prototype for other technologies such as the contraceptive pill or hormone replacement therapy, there remained a strong reluctance to import knowledge from the USA on its dangers and risks. Second, there was indifference to transgenerational side effects: even when the most visible effects of DES were finally acknowledged, there was a lack of consideration of the health of descendants; an inability to deem the knowledge of these repercussions as emancipatory or potentially empowering for the offspring. Third, regarding the health care of DES daughters, an important propensity to undone science is highlighted, with notable indifference to the risks of hormonalization of the female body, even on the part of activists. Thus, decades after it was last given to pregnant women, the shadow of DES still lingers as a failed reproductive health technology.

## Introduction

Diethylstilbestrol (DES), a synthetic oestrogen, has long been regarded as a ‘magic pill’, and in particular as a simple way to bring pregnancies to term, resulting in happy parents of a ‘healthy baby’. Synthesized by the English chemist Charles Dodds in 1938, DES began to be used in the USA in 1941. As Nancy Langston (2010) pointed out, it was the first drug to pass the newly created Food and Drug Administration (FDA) regulations at the time. From the 1940s onwards, DES was destined to cure not only the troubles of menopause, but many different diseases affecting all ages, from the young to the elderly. Among these conditions, miscarriage quickly became the most popular target, as ‘healthy babies’ symbolized the promise of DES use among pregnant women, although no consensus regarding its benefits had been reached within the academic community. Moreover, the ways of administering DES varied from one place to another, following different interpretations of the same ‘hormonal theory’. George and Olive Smiths, two Harvard physicians, theorized that failures of pregnancy might be due to low levels of oestrogen and were thus treatable with DES. This theory was so widely accepted that the results of one of the first randomized controlled trials in 1953, demonstrating the inefficiency of DES, were overlooked (Dieckman et al., 1953). While these researchers found no particular property in DES that could sustain pregnancy, millions of women in the USA and around the world were nonetheless administered it as one of the first reproductive health technologies.

In April 1970, Dr. Arthur Herbst from the University of Chicago published six case studies of a specific gynaecological cancer, clear cell adenocarcinoma (CCA), in young women aged 15–22 years (Herbst and Scully, 1970). The following year, the same team published a second article (Herbst et al., 1971) proving that these cancers had been caused by in-utero exposure to DES. For the first time, a clear and direct link was shown between taking a medicine during pregnancy and morbid effects on offspring after a considerable timeframe had elapsed. This publication was considered an alert, first at regional level, leading to investigations in various medical services, and ultimately led to national action with the FDA’s publication of a contraindication to the prescription of drugs to pregnant women in November 1971. This publication also motivated the creation of the first CCA cancer registry, which led to secondary analyses of patients’ maternal histories, the nature of their tumours and the treatments they were offered. Since then, the list of actual and potential side effects of in-utero exposure to DES has grown steadily to include infertility, significant prematurity, urogenital malformations, cancers, psychiatric disorders, eating disorders, etc. Moreover, the affected populations have grown to include ‘DES daughters’, ‘DES sons’ and even ‘DES grandchildren’.

The history of DES use in the USA can be summarized by a general lack of precaution and a series of unheeded warnings, until the drug was ultimately prohibited in 1971 (Langston, 2008). The drug’s trajectory in France repeated and exacerbated these tragic mistakes. Forty years after it was banned for pregnant women in the USA, in 2011, a survey by the French Agency for Drug Safety showed that many doctors were still ignorant of the effects of DES, especially on the offspring of the women who had taken the drug. Reproductive problems were cited by only half of doctors. Moreover, DES exposure was not searched systematically in cases of late miscarriage or in cases of a long history of miscarriages. The fieldwork undertaken by the present authors identified many DES daughters who recounted a long history of clinical problems before diagnosis. The aim of this article is to understand the sustained ignorance surrounding reproductive health knowledge, both within affected families and the entire medical profession, from an ignorance studies perspective (Gross and McGoey, 2015).

## Materials and methods

The history of DES in France had not been the subject of any academic work in the social sciences until 2010. This research, funded by the National Research Plan on Endocrine Disruptors, constitutes the first large-scale study conducted on this topic. It includes three parts: reconstructing the entire historical trajectory of DES in France from 1971; describing and analysing the medical and social experiences of people affected by DES, especially girls exposed *in utero*; and understanding the role of this experience on the treatment of endocrine disruptors, that is, chemicals interacting with hormonal systems at specific moments, causing cancerous tumours, birth defects and other developmental disorders. In order to respond to fulfil this research programme, the investigation involved multiple components and mixed methods.

First, exhaustive documentary research was performed, ranging from scientific literature to documents pertaining to the production of civil organizations, and even individual testimonies and blogs. Based on these elements, and particularly the list of scientists included in the scientific councils of the civil organizations ‘Réseau DES France’ (DES Network France, founded in 1994) and ‘Halte aux Hormones Artificielles pour les Grossesses’ (Stop Artificial Hormones for Pregnancy, founded in 2002), an initial list of actors to meet was established. This list was enriched as the interviews progressed, following a classic ‘snowball’ effect method.

Most of the key actors in this issue were still active at the time of the authors’ fieldwork (2010–2012). Thirty-one interviews were conducted and fully transcribed. These meetings with public health professionals, key clinicians and members of activist organizations were an opportunity to collect numerous documents (scientific articles, press cuttings, meeting minutes, reports, newsletters, etc.). For the period preceding electronic archiving of newspapers, microfilms from the ‘Bibliothèque Publique d'Information’ and press files of Science-Po Paris Library were used. In addition, audiovisual archives of the INA from 1980 to 2000 were used. This enabled the authors to verify that no articles on DES were published for the general public before 15 February 1983, and that this issue was only the subject of very limited media coverage before the trials of the 2000 s, with less than 100 articles from the period 1983–2000.

For the research dedicated specifically to the social experience of transgenerational disorders, civil organizations were first used as sources of recruitment in an informal way, by taking advantage of general meetings or symposia. Next, a more formal recruitment modality was used, by making a call for participation through the mailing lists of ‘Filles DES’ (DES Daughters, founded in 2003) and ‘Réseau DES France’. This led to 77 qualitative interviews (lasting between 1 and 4 h) with people affected by DES (17 mothers, three fathers, 48 daughters, two sons, four spouses of DES daughters, and three brothers and sisters of DES daughters).

One component of this research involved a comprehensive analysis of legal action surrounding DES-related cases, founded on interview data coupled with an analysis of 82 court decisions and observations at Nanterre court during hearings for ongoing cases of DES-related injuries (infertility, disability related to extreme prematurity) between victims and laboratory representatives. This part of the research demonstrated that while these legal proceedings concern individual cases, they contribute to a collective social and political experience (Fillion and Torny, 2015).

Contrary to the initial hypothesis that DES could have served as a precursor in the management of endocrine disruptors, it is shown that DES marked a failed precedent, with light shed on the different mechanisms producing ignorance of this issue. Environmental mobilizations and DES mobilizations in France long remained alien to each other, even after DES had been identified as the first endocrine disruptor in 1991 (Wingspread Conference, 1991). Three complementary processes to understanding the marginalization of this issue are analysed: lack of identification of exposed populations, minimal production and dissemination of knowledge, and long-term isolation. A previous article (Fillion and Torny, 2016) focused on DES as the first (retrospectively) known endocrine disruptor. The present article follows up on these results by going back to the main usage of DES as a reproductive health technology used to deliver ‘healthy babies’. In doing so, this article highlights the importance of ignorance in the experience of the sexual and reproductive health of DES-exposed persons, rather than pointing out questions of product liability, medical responsibility and drug regulation.

## Lost in translation: barriers to the import of knowledge

Since 1971, various groups have tried to mobilize around DES issues. DES activism was not an isolated effort, but was deeply inscribed within the greater women’s health movement (Bell, 2009). Even so, this activism had its own motives: advocating for the production of knowledge and for the recognition of DES patients as medical victims. By the end of the 1970s, its effectiveness led to DES victims receiving attention in the US legal sphere, through the development of jurisprudence within tort law to allow for their compensation (Schultz, 1990, Sheffet, 1983).

The French movement, in contrast, began much later and was more isolated: it was also led by women, but in no way constituted a feminist movement or even a women’s health organization. It only gained visibility in the 2000s through civil courts. Strong victories for victims were relayed in the media following decades of silence (Fillion and Torny, 2015). This discrepancy between mobilizations in France and in the USA stemmed from a decade of failed transnational propagation of knowledge produced in the USA. To understand this French delay, there is a need to go back 50 years and observe how France handled adverse drug effects at the time. Several key elements must be underlined. First, the French medical world was long dominated by clinical tradition, not by evidence-based medicine; scientific and moral authority was attributed to the clinician (Dodier, 2003). Second, pharmacovigilance was still in its infancy until the 1980s; the administrative and scientific control mechanisms for therapeutics were to be built later as coordinated entities (Urfalino, 2000). Finally, medicine was considered to be a matter for specialists: ‘laypersons’, patients especially, were not considered to be legitimate interlocutors in medical debates until they gained their place in the context of the acquired immunodeficiency syndrome epidemic (Barbot, 2002).

Yet, in the 1970s, Arthur Herbst, the doctor who proved the transgenerational effects of DES, was invited to make the transatlantic trip to present his results in France. There were few French doctors who subscribed to US medical journals at that time. Albert Netter, one of the ‘popes’ of French gynaecology in the second half of the 20th century, was an exception. In 1971, he created the ‘Journées de formation médicale continue’, an annual event of continual training for obstetrician-gynaecologists, and the following year invited Arthur Herbst to give a speech at this occasion. However, the majority of the professional elite who attended the event refused to believe in the possible deleterious effects of DES on their patients. One witness recollected the following:

Three great French gynecologists said: 'But of course, just because in one corner of the USA a large number of cancers have been found… [this doesn’t mean there is a problem] there can be a large number of factors [other than DES]' (RFI, 2011).

In addition, some physicians pointed out the differences in dosage or administration modalities in France and in the USA, notably the combined use of DES and progesterone to minimize the risks incurred by French patients.

A minority of the audience, however, considered the alert to be well founded, and reported these results in the French medical press, insisting on the need to stop all prescriptions to pregnant women and to monitor exposed girls with unexplained bleeding. These articles had little effect. A young obstetrician who had prescribed DES, Janine Henry-Suchet from AP-HP (Public Assistance-Paris Hospitals, university hospital centre of the Ile-de-France region, which includes some 40 public hospitals), decided to go a step further and call her patients back to her office. She suggested that they follow their daughters with a relatively simple and non-traumatic vaginal examination for early detection of possible CCA. Convinced of the benefits of such a clinical assessment, Dr. Henry-Suchet tried to mobilize her colleagues: she asked the French National College of Obstetricians and Gynaecologists to inform medical professionals systematically of this initiative. After a bitter negotiation, she finally obtained the postal addresses of members of the academic society and wrote to them herself. According to the present authors’ interview with Dr. Henry-Suchet, only three responded. When asked why she chose to pursue her investigations with so little support from her peers, she explained that, as a woman, her chances of obtaining the prestigious position of university professor-hospital practitioner were little to none, which certainly made her less visible, but also free from professional backlash. Thus, when knowledge from US research began to circulate in France in the early 1970s, it was given little consideration. Dr. Henry-Suchet therefore produced her own French data and published a paper based on her patients, showing dysplasia and vaginal adenosis in small infants exposed to DES contrary to infants exposed to ethinyl estradiol, another synthetic hormone, notably prescribed in cases of hypofertility at the time. However, most of the medical establishment still did not believe in DES problems: as ‘no cancer was being found in France’, the issue was deemed to be a US problem alone. This ignorance, which can be qualified as ‘strategic’, is present in other realms, to the point of ‘governing the world’ according to McGoey (2019). For example, Dedieu and Jouzel (2015) have made similar observations with respect to actors of occupational risk prevention in agriculture who are resistant to ‘uncomfortable’ knowledge about the dangers of pesticides: they consider the alerts as manageable risks that do not require a major revision of their conceptual approach and their practices. The present research points to a specific dynamic of strategic ignorance among French doctors regarding US health alerts related to drugs, partly informed by the conviction that French medicine is less influenced by the pharmaceutical industry, and more cautious in its prescriptions. Most French doctors considered that the US alert was not applicable to France – an argument that was later mobilized in cases of blood product contamination (Fillion, 2009) and hormone replacement therapy (Löwy and Gaudillière, 2006).

Publication of the first case of CCA in a young French girl in 1975 changed the situation. Like the first US cases, she had been exposed *in utero* to DES prescribed to her mother. This case occurred at a time when French pharmacovigilance was in its early stages. It was then organized around antipoison centres and, as a result, was mainly focused on acute and rapid effects. Adverse drug reactions were treated in the same way as accidental exposures to other toxic, chemical or biological agents. The very long-term and offspring effects of DES were therefore considered an oddity. Nevertheless, the publication of this cancer case was enough to lead the National Pharmacovigilance Commission to react.

At the time, the Commission was driven by a desire to reform information on adverse drug reactions, as public affairs had multiplied since the Stalinon precedent. This anti-staphylococcal drug, developed in the early 1950s, was sold without a prescription. Very quickly, serious health problems arose and numerous deaths occurred. This widely publicized affair was one of the first in France to highlight the weakness of drug safety controls (Bonah and Gaudillière, 2007). Although the Stalinon scandal initiated reforms in drug regulation, it only partially compensated for the structural weakness of drug safety. A new generation of medical pharmacologists at the end of the 1970s committed to granting pharmacovigilance a more robust institutional and scientific basis, based on the gold standard of randomized controlled trials. These young doctors were recruited for the creation of the Directorate of Pharmacy and Medicines in 1978, and became the elite of institutional pharmacovigilance in 1990–2000. However, as Urfalino (2000) has shown, this project, which began at the end of the 1970s, did not truly come to fruition until the creation of the ‘Agence du medicament’ (Drug Administration) in 1993. The history of DES allows insight into the slow process of transformation. Mandated by the Minister of Health and led by young pharmacologist Jean-Michel Alexandre, who would ultimately become a central figure in pharmacovigilance, the National Pharmacovigilance Commission aimed to standardize information on adverse drug reactions. The ‘Alexandre Commission’ proposed to include said information in the ‘Dictionnaire Vidal des médicaments’, a publication long financed by the pharmaceutical industry, sent free of charge to prescribers and often criticized for its advertising and lack of rigor. In 1976, DES ceased to be indicated for the prevention of miscarriage; in 1977, pregnancy was identified as a contraindication; finally, in 1978, the following was clearly instated: DES ‘is contraindicated in women who are pregnant or likely to become pregnant; vaginal adenoses and even vaginal cancer have been reported in pubescent girls and young women whose mothers had taken diethylstilbestrol or related estrogenic substances during pregnancy’. Thus, similar to the case of hormone pregnancy tests in Britain around the same time (Olszynko-Gryn et al., 2018), it took almost a decade to stop the use of a defective reproductive health technology. However, the lack of importation of US knowledge did not end there.

It was at this moment that a key figure in the French history of DES, Dr. Anne Cabau, intervened, whose work contributed heavily to publicizing the issue. She raised the question of multiple deleterious effects of DES, beyond CCA which had motivated the contraindication in 1977, and her work contributed to the first alerts launched to the public sphere. In the early 1980s, Dr. Cabau was a young gynaecological clinician who had completed her training in the USA and continued to consult the US medical literature. She was following young women at Béclère Parisian Hospital for infertility, and discovered that many of them had uterine abnormalities that reminded her of the US work on girls exposed to DES. She decided to investigate the offspring of women who had ingested DES during pregnancy. In 1981, Dr. Cabau used a mutual assurance journal to reach out to members and ask them about their offspring exposed *in utero*, both girls and boys. No cancer was documented in her corpus, and the young age of the vast majority of DES daughters did not enable her to identify fertility problems. However, the investigation showed an increase in the rate of cryptorchidism in DES sons. Dr. Cabau suggested vigilance and anticipation to the mutual assurance adherents: ‘if your daughter wants a pregnancy, an X-ray of the uterus is desirable to look for any abnormality’. At the same time, she published an international literature review on the subject, as well as her series of clinical cases of uterine malformations and reproductive disorders (Cabau, 1982).

In February 1983, Dr. Cabau's results were taken up by Dr. Escoffier-Lambiotte, head of the ‘Science and Medicine’ section of ‘Le Monde’, the most prestigious French daily newspaper, under the title ‘DES children: a monumental medical error’ (Le Monde, 1983). As soon as it appeared, this article was the subject of intense media coverage. Dr. Cabau was accused by a large number of colleagues of ‘sensationalism’ and personal advertising, which is forbidden by French law for doctors. The intense publicity surrounding this publication put strong pressure on health institutions and the medical profession. Both adopted a very reassuring tone in response: the Cabau study ‘would fortunately seem to indicate that the phenomenon is not on the same scale and severity as in other countries’ (French Health Ministry, Press Release, 16 February 1983).

Thus, over a decade later, the knowledge produced in the USA under the pressure of activists was first ignored, then imported by pioneers who read it and ultimately produced their own French knowledge on the subject, with a very limited audience. Although academic knowledge is supposed to be universal, it would seem that certain types of knowledge prove difficult to import, such as that of self-help practices in gynaecological health (Ruault and Rundell, 2016). It has been shown that the language barrier, the lack of reading of US literature, and the conception of medical practice as a national endeavour have limited the circulation of knowledge. Specific national cultures within narrow medical segments tend to persist over long periods of time, before being called into question by international literature, as shown in the case of progestins prescribed to premenstrual women (Löwy and Weisz, 2005). Beyond these cognitive factors, one must also consider the widespread indifference to the transgenerational issue, and reluctance to inform patients of their own exposure to DES.

## No more use of past knowledge: indifference to the transgenerational question

At the time of the public discussion of DES in the national press, in March 1983, the National Pharmacovigilance Commission did not mention the increased risk of late abortions or ectopic pregnancies, although these were already documented in the US literature. On the contrary, Prof. Royer specified that exposed DES daughters were mostly succeeding in carrying their pregnancies to term, saying, ‘we must remain reasonable, “big” problems are essentially a rise of miscarriage risks during pregnancy and some rare vaginal cancer cases’ (Royer, 1983). Far from recommending hysteroscopy, the Commission advised physicians to perform an annual Pap smear ‘in case of anxiety on the part of the family or the young woman’ (Le Panorama du Médecin, 1983). This discourse shows that the information of women and public debate regarding DES were conceived as a source of ‘panic’ and never as one of empowerment.

In June 1983, a group of researchers commissioned by the French Institute of Health and Medical Research conducted a study and found that 200,000 women had been treated with DES in France, and 160,000 living babies had been exposed *in utero*. Based mainly on the US literature, this group, led by Alfred Spira, an epidemiologist, issued a series of recommendations outlining both how to inform and support these patients, and how to train healthcare professionals. However, these recommendations remained largely unheeded. Furthermore, there existed a shared belief among health authorities that healthcare professionals were aware of DES issues, meaning their patients would be as well. Thus, the answer to a parliamentary question on the right to information of patients exposed to DES, addressed in the summer of 1983, depicted an almost ideal situation of knowledge and information:

With regard to preventive measures, it appears that gynaecologists and specialists in our country are well aware of the issue and have been since 1971, and that the degree of alert of the entire medical profession seems to be sufficient. Nevertheless, the National Pharmacovigilance Commission has considered appropriate to reformulate the recommendations defining the conduct to be followed in respect of these patients, and all the major daily newspapers and the professional press have widely reported on them. The public can therefore be considered well informed (French Parliament, 1983).

The present investigation shows the exact opposite situation: ignorance of doctors was disregarded, and most DES families did not know of their condition or of the optimal medical care. The authors collected numerous requests for information from patients and exchanges of correspondence with their doctors at that time. The doctors themselves, however, did not necessarily have access to this knowledge, for it was not disseminated systematically in the French medical realm. Likewise, publications on DES failed to recommend any particular surveillance. Whether due to a lack of specific interest in the subject, insufficient training or the absence of collective vigilance, a massive oversight was thus produced and sustained within French medicine. This oversight was particularly demonstrated by three unsuccessful attempts to mobilize the medical profession between 1988 and 2003. Each time the French DES context was revisited, it was noted that doctors were unaware of the problem or did not inform their patients.

In January 1988, the Ministry of Solidarity, Health and Social Protection brought a group of 11 clinicians to draw up an informational brochure, under pressure from DANES 45, the first civil organization dedicated to DES created in France in 1986. While confirming the massive ignorance of the problem on the behalf of health professionals, this group of experts insisted on the uselessness of specialized reference centres and the need to maintain a strictly professional information circuit: only two participants (out of 11) considered the brochure to be absolutely necessary for the public, while the others decided it ought to remain within medical circles (French Health Department, 1988). Five years after the revelation by ‘Le Monde’ of this major health problem, public information and specialized care were again judged as problematic. This brochure, published by the French Centre for Health Education (CFES), was sent discreetly to physicians, midwives and obstetrician-gynaecologists in 1989.

A few colleagues, mainly obstetrician-gynaecologists, joined the two clinicians who had advocated for the public dissemination of this brochure. These were practitioners dealing with the adverse clinical effects of DES within their caseload. The interviews conducted with them show their surprise and deep disappointment regarding the inertia of the medical profession and health institutions. These practitioners attribute their alliance to a desire to create a working group that would not have to repeatedly demonstrate the deleterious effects of DES, and that could devote its energy to finding concrete answers to their patients' problems. This group began to participate in collective information meetings targeted at DES families. While there were only about 10 members, they nonetheless made a long-lasting commitment. This led them to get closer to ‘Info DES’, the second civil organization of French DES victims, created in 1990. When ‘Info DES’ organized a European awareness week on DES in France, these clinicians widely relayed the need to ‘screen’ the 80,000 girls exposed. Recommendations, such as the realization of a systematic hysteroscopy when a DES girl wants to have a child, were thus communicated by the medical and general press in April 1992. At the same time, the ‘Direction générale de la santé’ (General Directory of Health) disseminated the CFES brochure (beyond medical circles), accompanied by a letter from the Director General of Health.

Despite these campaigns, in the early 2000s, ‘Agence française de sécurité sanitaire des produits de santé’ (AFSSAPS) (French Agency for the Safety of Health Products) was alerted by the third organization created by DES-affected persons in 1994, ‘Réseau DES’, about the general ignorance of the problems associated with DES for the second generation of victims. In the meantime, all DES daughters had entered reproductive age and some of them were encountering major obstetric problems, described regularly in the US literature since the end of the 1970s. Adhering to the recommendations elaborated by ‘Réseau DES’ and the group of mobilized gynaecologists, AFSSAPS sent a letter to all general practitioners and obstetrician-gynaecologists at the beginning of 2003. Although it listed practices already described in previous information campaigns, their modalization changed. Whereas in 1988, the CFES brochure had stated that ‘identification is difficult but can be practiced in certain circumstances’, the AFSSAPS letter now indicated that ‘it is necessary to think about in utero exposure when interviewing all woman born before 1977′ or even that ‘in utero exposure to DES must be systematically investigated’ (AFSSAPS, 2003).

The consequences of this ignorance on the second generation of DES victims also led to poor public policies, as is particularly evident in the long history of maternity leave, specifically for DES daughters. As early as the late 1980s, organizations demanded maternity leave as soon as pregnancy posed as a reason to stop working. In addition to the expected financial and social benefits of maternity leave, the main motivation for this demand was based on clinical knowledge: women prone to spontaneous abortions throughout their pregnancy would have a better chance of carrying them to term if they were granted leave. The organizations received numerous refusals, particularly from Social Security authorities, which feared that this provision would set a precedent and therefore referred them to a joint care facility. It was only at the end of 2004, following the initiative of a member of parliament, that a legislative provision was passed. When the implementing decrees were signed in 2006 for private sector employees and in 2010 for those in the civil service, most DES daughters had already passed childbearing age. Moreover, these decrees remained little known, and when interviewed, ‘DES-specialized’ obstetricians regularly complained that they themselves had to inform Social Security of the existence of this provision. In practice, this signified miscarriages in the third trimester, very premature babies and very handicapped DES grandchildren still happened in the 2010s.

Although some gynaecologists are effectively informed today, the difficulties faced by these informational campaigns explain why many are still not fully aware of the DES issue. The authors’ interviews with DES families, as well as the testimonies collected by patient associations, found that obstetrician-gynaecologists frequently declare that they have ‘never seen DES daughters’, or that women having suffered from typical DES symptoms for long periods of time discovered the cause of their problems through television programmes (e.g. the documentary ‘Without Principle or Precaution’, broadcast in 2002) or the publicity given to court rulings. The choice to disseminate knowledge to health professionals alone has made it impossible for the exposed populations to know of their status and to be vigilant about the risks they run.

## The reproductive health of DES daughters: cases of undone science

In addition to the disqualification of knowledge examined in the first part of this paper, and the non-mobilization (or very partial mobilization) of knowledge examined in the second part, the French DES case illustrates a third modality of production of ignorance: ‘undone science’ (i.e. knowledge that could have been produced but was not) (Frickel et al., 2010). Local clinical knowledge and know-how are included in this process of undone science which, due to lack of research, could not reach the status of scientifically proven knowledge.

The USA and the Netherlands both offer interesting examples of a-posteriori cohort construction: the DESAD Project (DES and Adenosis project) initiated in 1974 in the USA is an observational study of DES daughters based on 4830 identified mothers. From 1992, the Dutch set up a self-declaration register of women who had been prescribed DES: from here, cohorts of their descendants were constructed. The US cohort documented the increased risk of cancer in DES mothers and daughters (see, in particular, Palmer et al., 2006, Titus-Ernstoff et al., 2006). The Dutch cohort documented the increased risk of hypospadias in DES sons (see, in particular, Brouwers et al., 2006). Meanwhile, French health institutions have never initiated an epidemiological cohort of this type.

In France, there has been a lack of attention to the transgenerational consequences of DES, which, beyond limiting knowledge dissemination abroad and accounting for unndone epidemiological research, has marginalized the clinical knowledge produced in France. This has deprived the medical community and patients of proven and shareable knowledge. A small group of clinicians has thus been left to invent and adapt practices to deal with the specific troubles of DES daughters. From the beginning of the 1990s, under the leadership of ‘Info-DES’, these doctors regularly organized information meetings within their hospital structures. The European Mobilization Week of 1992 marked an opportunity to publicize the research discussed in these organized meetings. However, this publicity was always initiated and performed by DES associations and, as such, did not exhibit any official legitimacy. In 1996, Dr. Cabau decided to open volunteer specialized consultation at the Saint-Vincent-de-Paul Hospital in Paris, without any support from the hospital’s administrative board. Outside of Paris, specialized places were even less visible and one of the main roles of the regional correspondents of ‘Réseau DES’, which succeeded ‘Info DES’ in 1994, was to refer women to these structures. Even today, these consultations are not reported on the AP-HP website and no budget is devoted to them. However, the few DES-involved doctors have not ceased to develop and adapt specific knowledge and know-how, in accordance with the classic process of clinical knowledge development, based on continual experience and accumulated cases (Dodier, 2003). They particularly concern medically assisted reproduction techniques, pregnancy monitoring and an original treatment for CCA.

Sylvie Epelboin, a specialist in hypofertility and medically assisted procreation (MAP) who was first employed in Saint-Vincent-de-Paul Hospital and then in Bichat Hospital, has welcomed an increasing number of exposed girls to her active file, a proportion that she estimated at 25% in 2010. Aware of the fact that exposure to hormones had been deleterious for these women and that the massive use of hormones in MAP procedures has not always been evaluated scientifically, she has adopted a cautious attitude. Proceeding with this attitude in the event of successful in-vitro fertilization, she opted for the implantation of a single embryo in DES daughters with less well-developed and less well-vascularized uteruses. Although Dr. Epelboin attempted to systematically document the prevalence of girls exposed in MAP consultations, she was unable to obtain a DES item to be added to the standard questionnaires sent to women in all specialized centres. She also initiated research protocols based on her active file, but these have not been sufficiently expanded to gain statistical significance. Although she has continued to communicate at specialist congresses, to the point where she has been ironically nicknamed ‘Mrs DES’ in the MAP community, and many patients deemed ‘difficult’ candidates for IVF have been referred to her department, she has published little about her adaptive techniques. Her specific reproductive treatment has therefore not been evaluated in the sense of evidence-based medicine (Weisz, 2005).

Pregnancy care techniques have followed the same trajectory, that is the construction of highly specialized and empirical knowledge, without wide dissemination or peer review evaluation. Anne Cabau and Michel Tournaire – one of the obstetricians from AP-HP committed in the clinical follow-up of DES daughters and in ‘Réseau DES’ – have defended the use of cervical cerclage as a preventive measure against miscarriage. This surgical technique involves placing a wire around the cervix to keep it closed until the end of the eighth month of pregnancy. In France, cerclage was generally recommended after several adverse events. Doctors who prescribed it as a first-line treatment for DES daughters were therefore rare. As M. Tournaire explained to us:

So this woman, […] I told her: 'in France, some people expect three accidents'. And she said: ‘it's me’, ‘why?’, ‘because I had three miscarriages at 19, 21 or 20 weeks, and I was told: next time, we're going to give you a cerclage’… And so, I don’t wait for them to have their accidents [before acting] (Interview 1, 2010).

There has been long-lasting controversy surrounding the systematic use of this preventative technique and its effectiveness among DES daughters. Its adoption is therefore dependent on the gynaecology services through which women are medically accounted. The adaptation of this technique is delicate, especially given the small size of the cervix in these patients. In Saint-Vincent-de-Paul Hospital, only Prof. Tournaire practised this type of cerclage, and it was only when he retired in 2011 that the department grew concerned about passing on his knowledge to other physicians.

It is in the treatment of CCA that the most original clinical knowledge has been developed in France, at the Institut Gustave-Roussy (IGR) within Alain Gerbaulet's team of oncologists who treated approximately 60 DES daughters with cancers. Unlike the USA, which opted for mutilating surgeries for the purpose of eradication or prevention, the IGR team developed brachytherapy, a very localized form of radiotherapy, to preserve the patient's organs as much as possible, combined with conservative surgery. The treatment was personalized, thanks to imprints and the use of miniaturized radioactive sources adapted to each patient, depending on her anatomy and the location of tumours. Preservation of the ovaries, vagina and uterus was essential for very young patients who may not have completed their growth and, more generally, functioned to retain the possibilities of a sexual and reproductive life. This work was published in English and also received some publicity when a former patient of Dr. Gerbaulet gave birth to a child in 1991. This resulted in articles in the popular, women's and general press alike. Since then, a few other pregnancies have been completed among nearly 60 patients treated at IGR (Magné et al., 2012).

Other techniques such as enlargement hysteroplasty, a surgical procedure for reconstruction of the uterus, complete the portrait of essential clinical knowledge in the field of reproductive health that has not been recognized officially. The quasi-exclusive route of entry was that of organizations, which propagated contact details for services, organized meetings, published interviews with doctors, and so forth. From the end of the 1990s onwards, this activity was formalized with the creation of a scientific council within ‘Réseau DES’ and the production of clinical information brochures. Accordingly, women who were unaware of their exposure, the risks it involved, or the existence of organizations working on these issues were most often condemned to a long diagnostic and clinical wandering, or even to inappropriate and iatrogenic treatments. Clinicians favoured papers in symposia, as international publications remained rare. Knowledge about exposed patients was fragmentary. A book of clinical articles devoted almost exclusively to DES daughters was published in 1991, and a revised and expanded version, with less confidential distribution, was made in 2007 (Blanc and Florence, 2007). The first symposium dedicated to the issue was held in 2011 at the initiative of ‘Réseau DES’.

These clinicians have therefore been highly involved in treating DES daughters, but they have not considered dangerous hormonal treatments as a whole, contrary to activists depicted in Susan Bell’s book (2009). In the USA, the DES crisis did not lead to massive rejection of all hormones, but fuelled major criticism of the hormonalization of women’s bodies, especially in the context of contraceptive practices. The very marginal criticism in France of the hormonalization of women would need to be studied with regard to the specificities of the French feminist movements and its alliances with the medical profession, in accordance with Ruault’s work (2017) on abortion techniques. In particular, the important connections between feminist mobilizations and medical gynaecology in the 1970s and 1980s should be further illuminated. This original medical segment, unique to France, was mainly composed of women who provided gynaecological follow-ups for their patients, from the first requests for contraception to the treatment of menopause.

French clinicians were concerned that DES daughters may benefit from adapted contraception. The increased risk of ectopic pregnancy discouraged them from prescribing intra-uterine devices and guided them in favour of hormonal contraception. The frequent problems of hypofertility and the use of MAP also argued in favour of hormones in various procedures, such as ovarian stimulation and uterine re-implantation. In 2003, the creation of a new association, ‘Les Filles DES’, whose action was very much oriented towards access to parenthood, reinforced this course of action. Hormone replacement therapies prescribed in the context of menopause were no longer the object of suspicion for DES daughters: the call for vigilance came late in the early 2000s, as it did for the general population. For both health professionals and patients, DES was therefore not perceived in France as a tragedy that prompted critical work on the ‘hormonalization of women’ like that carried out in the USA since the 1960s (Krieger et al., 2005).

## Conclusion: three generations to fight ignorance

French DES mothers, estimated to be 200,000 in number, did not know they were taking risks by ingesting this drug. Even after 1971, they had little information about the dangers of DES. Their daughters rarely knew about their condition, and were left to depend on a medical profession with little training, especially when it came to reproductive health, for help. Unless their birth had been completely reshaped by the drug, DES grandchildren rarely knew of their medical heritage. Whereas in the USA, the DESAD cohort was set up in 1974, no such instrument has been promoted by the public authorities in France, and even the CCA cancer registry, recommended by Alfred Spira’s team, has not been set up. Despite ongoing criticism and clear failures to identify health problems, which point to ‘strategic ignorance’ (McGoey, 2012), randomized clinical trials and large cohorts remain the dominant forms of evidence for public authorities and the medical profession. These medical tools are time-consuming and costly to build and maintain, which accounts for a failure to do so, at the risk of suggesting that no new knowledge can or should be generated.

For the first time, more than 40 years since Arthur Herbst’s visit to Paris, robust epidemiological knowledge on French DES mothers and their offspring began to be produced, in order to break the cycle of ongoing ignorance. In fact, in 2013, ‘Reseau-DES’ launched a major case-witness investigation (Study ‘DES 3 Generations’ National survey on the consequences of DES, Réseau DES, 2014), with the support of a mutual insurance company and the newly named National Drug and Health Safety Agency to fight systemic ignorance. The survey focused on the undesirable effects suffered by three generations of exposed populations. The survey was premised on a need for new epidemiological results. For one, controversy regarding the divergent results of the US and Dutch cohorts, particularly with respect to the excess risk of breast cancer in DES daughters, had permeated the French civil organizations’ milieu. Furthermore, an article on the excess risk of genital malformations in grandsons, produced from French data, had been widely addressed in the general press. Although the survey has been financed by ANSM, the design of the 3-Generations DES cohort has remained under the supervision of ‘Réseau DES’ and its clinical allies. The modalities of this research confirm the usual process of knowledge production about the side effects of DES in France: it has always been carried out under the pressure of organizations, as opposed to that of the medical profession or public health institutions. The involvement of the agencies in charge of the drug remains marginal, in line with a history of limited pharmacovigilance and consequent registration or reporting systems. What is at stake here is therefore a true evidence-based activism approach (Rabeharisoa et al., 2014), where activists use the techniques and vocabulary of evidence-based medicine to produce knowledge where once was undone science.

Despite a political context different from that of West Germany described by Birgit Nemec and Jesse Olszynko-Gryn (in this issue), some processes of production and maintenance of ignorance are shared between the DES history and that of Duogynon: undone science, disqualification of whistleblowers, disqualification of epidemiological studies that do not fit with gold standards, calls for precautionary behaviours addressed to doctors by public authorities that remains unheard, weak participatory culture until recently, etc. In the case of DES, in order to cope with such long-lasting ignorance, based on the disqualification (or at least weak recognition) of local clinical knowledge and the disqualification of patients’ and exposed persons’ information accused to be a source of ‘panic’, associations, families and their clinical allies have developed a variety of evidence-based activism strategies. The silence that has characterized the history of DES for more than 30 years has been regularly attributed to the ‘guilt’ of mothers who absorbed the drug which has impacted their offspring. On the contrary, this research shows that it is the people directly concerned and their organizations which have multiplied private and public interventions, testimonies, the sharing of experience, documented accounts, educational brochures, etc. in order to spread knowledge. As shown, these actors have largely failed in their quest for medical recognition, and it was ultimately judicial strategies that secured their status as DES victims and the associated social recognition (Fillion and Torny, 2015). Legal action has been decisive in re-evaluating and politicizing the history of DES in France: DES has shifted from being seen as an isolated problem of the past to an emblematic healthcare crisis. Indeed, the courts have made it possible to publicly objectify a health and social problem, and have contributed to the establishment of new rights through jurisprudence by imposing an obligation of vigilance on pharmaceutical companies. DES now constitutes a precedent for other new cases, notably those involving treatments specific to women: third-generation pills, antidepressant drugs prescribed to pregnant women, hormone replacement therapy, etc. As in the case of oestrogen therapies in the USA decades earlier (Seaman, 2011), combatting the production of ignorance required that judges be convinced of victims’ illnesses, and not just doctors or epidemiologists.

## Declaration

The author reports no financial or commercial conflicts of interest.
